# Accouchement chez la primipare à Lubumbashi: pronostic maternel et périnatal

**DOI:** 10.11604/pamj.2017.28.77.13712

**Published:** 2017-09-27

**Authors:** Roger Munan, Yves Kakudji, Joseph Nsambi, Olivier Mukuku, Amani Maleya, Xavier Kinenkinda, Prosper Kakudji

**Affiliations:** 1 Département de Gynécologie-Obstétrique, Faculté de Médecine, Université de Lubumbashi, République Démocratique du Congo; 2 Institut Supérieur des Techniques Médicales de Lubumbashi, République Démocratique du Congo

**Keywords:** Primiparité, accouchement, pronostic maternel et périnatal, Lubumbashi, Primiparity, childbirth, maternal and perinatal prognosis, Lubumbashi

## Abstract

**Introduction:**

La parturition des primipares est associée à de nombreuses complications et par conséquent, elles sont considérées comme étant à haut risque en raison des préoccupations maternelles et fœtales. La présente étude s'est fixé comme objectifs de déterminer la fréquence d'accouchement chez les primipares dans notre milieu, d'identifier les facteurs associés à l'accouchement par césarienne et d'évaluer la morbi-mortalité maternelle et périnatale lors de l'accouchement chez la primipare dans la ville de Lubumbashi.

**Méthodes:**

C'était une étude transversale analytique des accouchées d'une grossesse monofœtale de Décembre 2013 à Mai 2014 dans 10 maternités de référence à Lubumbashi. Les accouchées primipares ont été comparées aux multipares. Les paramètres sociodémographiques maternels, la morbi-mortalité maternelle et périnatale ont été analysées. L'odds ratio et son intervalle de confiance ont été calculés. Le seuil de signification a été fixé à une valeur de p<0,05.

**Résultats:**

La fréquence de la primiparité était de 19,9%. Comparativement aux multipares, les accouchées primipares étaient significativement adolescentes (OR = 11,27 (7,98-15,91)), élèves/étudiantes (OR=5,61 (3,33-9,45)) et vivaient seules (OR = 7,62 (4,36-13,30)). Les facteurs de risque associés à l'accouchement par césarienne chez les primipares étaient l'évacuation obstétricale (OR = 9,69 (4,75-19,74)), le manque de suivi de consultations prénatales (OR = 2,57 (1,32-5,01)), la taille ≤ 150 cm (OR = 2,42 (1,04-5,65)), la hauteur utérine >34 cm (OR=2,33 (1,32-4,10)) et la mal présentation fœtale (OR = 6,37 (2,92-13,87)). S'agissant du pronostic maternel, nous avons observé que la pression artérielle élevée (OR = 1,91 (1,32-2,74)), la mal présentation fœtale (OR=1,95 (1,16-3,17)), le recours à l'utilisation d'ocytociques (OR=2,03 (1,64-2,52)), la césarienne (OR=2,04 (1,47-2,83)), l'épisiotomie (OR = 11,89 (8,61-16,43)) et l'éclampsie (OR = 4,21 (1,55-11,44)), étaient significativement associées à la primiparité. Les taux de score d'Apgar déprimé à la fin de la 5^ème^minute (OR = 1,55 (1,03-2,32)) et de décès en période néonatale précoce (OR = 1,80 (1,08-2,98)) étaient significativement plus élevés chez les primipares que chez les multipares.

**Conclusion:**

Cette étude montre que l'accouchement de la primipare reste un problème obstétrical à Lubumbashi. D'où l'amélioration de la santé du couple mère-enfant lors de l'accouchement chez la primipare passe par l'élaboration des protocoles de prise en charge des accouchements adéquats.

## Abstract (en)

**Introduction:**

Childbirth in primiparous women is associated with many complications and, therefore, primiparous women are considered high risk due to maternal and fetal concerns. This study aims to determine birth rate in primiparous women in our environment, to identify factors associated with delivery by cesarean section and to assess maternal and perinatal morbi-mortality from childbirth in primiparous women living in Lubumbashi.

**Methods:**

We conducted a cross-sectional, analytical study of singleton births in 10 referral maternity hospitals in Lubumbashi over the period December 2013-May 2014. Primiparous births were compared to multiparous births. Maternal sociodemographic parameters as well as maternal and perinatal morbi-mortality were analyzed. The odds ratio and its confidence interval were calculated. Threshold significance level was set at p < 0.05.

**Results:**

Primiparity rate was 19.9%. Compared to multiparous births, primiparous births were mainly observed in adolescents (OR=11. 27, (7.98-15.91)), in students (OR = 5.61 (3.33-9.45)) and in women living alone (OR=7.62 (4.36-13.30)). Risk factors associated with delivery by cesarean section in primiparous women included obstetric evacuation (OR = 9.69 (4.75-19.74)), the lack of prenatal monitoring (OR=2.57, (1.32-5.01)), size ≤ 150 cm (OR = 2.42 (1.04-5.65)), uterine height > 34 cm (OR = 2.33 (1.32-4.10)) and malpresentation (OR = 6.37 (2.92-13.87)). With regard to maternal prognosis, we observed that high blood pressure (OR = 1.91 (1.32-2.74)), malpresentation (OR = 1.95 (1.16-3.17)), oxytocin use (OR = 2.03 (1.64-2.52)), cesarean section (OR = 2.04 (1.47-2.83)), episiotomy (OR=11.89 (8.61-16.43)) and eclampsia (OR = 4.21 (1.55-11.44)) were significantly associated with primiparity. The rates of low 5th minute Apgar score (OR = 1.55 (1.03-2.32)) and of deaths occurred during early neonatal period (OR=1.80 (1.08-2.98)) were significantly higher in primiparous women than in multiparous women.

**Conclusion:**

This study shows that primiparous birth is a problem in Lubumbashi. Hence improvement in mother-child care during primiparous childbirth includes the development of protocols for adequate management of childbirths.

## Introduction

Dans toutes les sociétés, l'accouchement est vécu comme un heureux évènement mais aussi comme une angoisse, car on ne connaît pas le dénouement, la vie ou la mort. Une primipare est une femme qui a une seule expérience d'accouchement [1]. Une femme portant sa première grossesse commence une nouvelle vie et il est un moment crucial dans sa carrière obstétricale que les performances obstétricales ultérieures dépendront de la façon dont la première grossesse sera gérée [2]. L'accouchement chez une primipare est souvent vu avec anxiété non seulement par la gestante qui passe par l'expérience pour la première fois mais aussi par sa famille et son accoucheur. La parturition des primipares est associée à de nombreuses complications et par conséquent, elles sont considérées comme étant à haut risque en raison des préoccupations maternelles et fœtales [2]. La période péri-partale a un risque plus élevé de mortalité pour le couple mère-enfant. On estime que 42% des 535 900 décès maternels annuels sont liés à l'accouchement [3]. En période périnatale, des décès étaient dans la même étude étroitement liés à la mort de 1,02 million de nouveau-nés pendant le travail et de 904 000 décès néonatals liés à l'accouchement [3].

L'identification au cours de consultations prénatales et avant le travail des femmes à risque de dystocie et le transfert en temps opportun à un hôpital de niveau supérieur pour l'accouchement est l'une des stratégies visant à réduire la morbidité et la mortalité maternelles et périnatales [4]. Le risque intra-partum est fondé principalement sur l'histoire obstétricale passée qui fait défaut chez toutes les primipares. Il existe des différences fondamentales entre le travail normal chez une primipare et celui d'une multipare. Comparées aux femmes multipares, les primipares sont plus susceptibles de développer des anomalies du travail qui nécessitent une intervention [5]. De nombreuses études ont montré que les premières grossesses sont à risque accru de complications pendant la grossesse, le travail et l´accouchement et si elles ne sont pas correctement gérées, ces complications peuvent entraîner une augmentation de la morbidité et de la mortalité du couple mère-enfant [6]. Les complications signalées sont notamment les troubles hypertensifs de la grossesse, l'accouchement prématuré et le faible poids à la naissance associé, les anomalies du travail, le risque accru d'accouchements par césarienne, les psychoses puerpérales, le taux accru d'hospitalisations néonatales et l'augmentation des décès périnatals [7–10]. Ces risques sont encore accrus chez les primipares dans les pays en développement en raison de la pauvreté, de l'utilisation insuffisante des soins prénatals, du manque d'installations optimales de surveillance du travail, du manque d´infrastructures et de dotation en personnel hospitalier qualifié, des pratiques socioculturelles défavorables.

Tout au long de nos recherches, nous nous sommes rendu compte qu'il existe très peu de travaux consacrés à cette entité en République Démocratique du Congo (RDC) en général et à Lubumbashi en particulier, aucune étude sur le sujet n'a été publiée jusqu'à ce jour. C'est dans ce contexte que s'inscrit ce présent travail qui s'est fixé comme objectifs de déterminer la fréquence d'accouchement chez les primipares dans notre milieu, d'identifier les facteurs associés à l'accouchement par césarienne ainsi que d'évaluer la morbi-mortalité maternelle et périnatale lors de l'accouchement chez la primipare dans la ville de Lubumbashi, RDC.

## Méthodes

**Figure 1:**
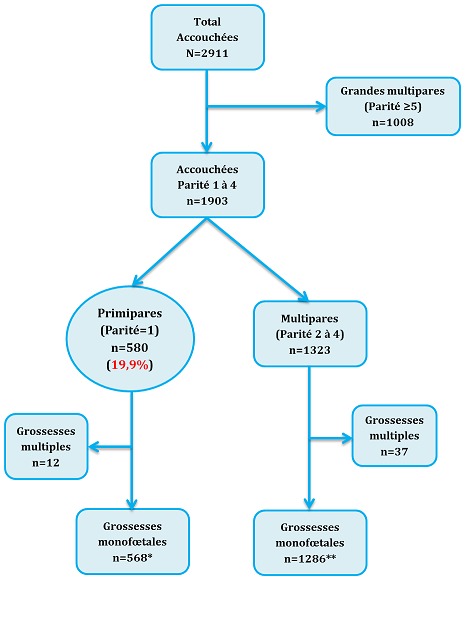
Distribution des accouchées enrôlées dans l’étude

**Type, période et population d'étude:** Il s'agit d'une étude transversale analytique menée sur la période allant du 1^er^ décembre 2013 au 31 mai 2014. Au cours de cette période d'étude, nous avons enregistré tous les accouchements réalisés dans les maternités des 10 hôpitaux généraux de référence (HGR) de la ville de Lubumbashi en RDC (hôpital militaire de Ruashi, Cliniques Universitaires, hôpital Jason Sendwe, HGR Katuba, HGR Kenya, HGR Kamalondo, HGR Kisanga, HGR Kampemba, hôpital Gécamines-Sud et hôpital SNCC). Ces hôpitaux sont répartis dans les 7 communes que compte la ville de Lubumbashi. Toutes les femmes qui se sont présentées dans ces formations sanitaires choisies pour un accouchement ont été incluses de manière consécutive et exhaustive dans l'étude quel que soit le lieu de suivi de la grossesse. Au total, 2911 accouchements ont été enregistré dont 568 ont concerné des primipares avec grossesses monofœtales ayant constitué le groupe de cas. Ces accouchées ont été comparées à celles des multipares (ayant une parité allant de 2 à 4) qui formées le groupe de témoins dont l'effectif était de 1286. La Figure 1 donne la distribution des accouchées enrôlées dans l'étude. La parité (nombre de grossesses ayant atteint au moins 22 semaines d'aménorrhée à l'accouchement) a été considérée comme variable dépendante et elle était enregistrée après l'accouchement.

**Variables d'étude:** Les caractéristiques sociodémographiques maternelles, les paramètres en rapport avec la morbi-mortalité maternelle et périnatale ont été recueillis par le personnel effectuant habituellement l´accouchement dans les sites d´enquête. Un entretien a permis de recueillir les caractéristiques sociodémographiques de la patiente ainsi que les antécédents obstétricaux. Une fiche d'enquête individuelle avait été élaborée à cet effet et la recherche de données complémentaires a été réalisée dans le dossier obstétrical.

**Caractéristiques sociodémographiques, anthropométriques et suivi de grossesse:** Âge de l'accouchée, niveau de scolarité, situation matrimoniale (sur déclaration de l'accouchée), profession, taille, suivi de la grossesse.

**Paramètres en rapport avec la parturition, la morbidité et la mortalité maternelles:** Mode d'admission, pression artérielle à l'admission, hauteur utérine (mesurée par un mètre ruban inextensible à l'admission), état des membranes fœtales à l'admission, terme de la grossesse à la naissance, mode d'accouchement, indications de la césarienne, présentation fœtale, utilisation de la solution ocytocique (au cours du travail d'accouchement pour redynamiser celui-ci), épisiotomie, notion de transfusion (nous avons noté si l'accouchée était transfusée en période péri-partale ou non), éclampsie, lésions de parties molles (réunissaient les déchirures cervicale, vaginale et périnéale) et issue maternelle (vivant ou décédé). Les indications de la césarienne ont été classées selon le type d'indications (dystocie mécanique, dystocie dynamique et autres indications “non dystociques”) et selon l'origine d'indications (maternelles, fœtales, annexielles et mixtes).

**Paramètres en rapport avec les nouveau-nés:** Poids de naissance, score d'Apgar (côté à la fin de la 5^ème^minute après l'extraction du fœtus), issue périnatale. La mortalité périnatale précoce concernait les décès des nouveau-nés après la naissance et avant la sortie de l'hôpital.

**Analyse des données:** La parité est considérée ici comme variable dépendante et les paramètres en rapport avec la morbi-mortalité maternelle et périnatale constituent les variables indépendantes. Les caractéristiques sociodémographiques, la taille, le suivi de la grossesse et la morbi-mortalité maternelle et périnatale des primipares ont été comparées à celles des multipares. Le test de χ^2^ corrigé de Yates ou le test exact de Fisher ont été utilisés pour comparer les fréquences. L'odds ratio (OR) a été calculé et présenté avec ses limites dans l'intervalle de confiance à 95% (IC à 95%) et le seuil de signification a été fixé à p < 0,05. Les analyses ont été réalisées à l'aide du logiciel Epi Info 7.2.

**Considérations éthiques:** Un consentement libre et éclairé de toutes les personnes impliquées dans cette étude a été obtenu verbalement. L'anonymat a été respecté. Les autorisations de Médecins Directeurs ont été préalablement obtenues.

## Résultats

Sur un total de 2911 accouchées consécutivement enregistrées au cours de la période d´étude, nous avons répertorié 580 primipares, soit une fréquence de 19,9% (Figure 1). Le Tableau 1 présente les caractéristiques sociodémographiques des primipares à Lubumbashi. Comparées aux multipares, l'analyse statistique montre que les primipares ont 11 fois plus de chance d'être plus jeunes (moins de 20 ans) (OR = 11,27 (7,98-15,91)), ont 7 fois plus de chance d'être seules (OR = 7,62 (4,36-13,30)), ont 5 fois plus de chance d'être des élèves/étudiantes (OR = 5,61 (3,33-9,45)). Par ailleurs, nous n'avons pas observé de différence statistiquement significative entre les primipares et les multipares en ce qui concerne le niveau de scolarité et le suivi prénatal (p > 0,05). S'agissant de la morbi-mortalité maternelle (Tableau 2), par rapport aux multipares, nous constatons que les primipares présentaient un risque significativement élevé d'avoir une pression artérielle élevée à l'admission (OR = 1,91 (1,32-2,74)), d'avoir un fœtus en mal présentation (OR = 1,95 (1,16-3,17)), de recours à la solution ocytocique au cours de la parturition (OR = 2,03 (1,64-2,52)), d'accoucher par césarienne (OR = 2,04 (1,47-2,83)), de subir une épisiotomie (OR=11,89 (8,61-16,43)) et de faire une éclampsie (OR = 4,21 (1,55-11,44)). Quant au mode d'admission, à l'âge gestationnel, à l'état des membranes fœtales à l'admission, à la survenue des lésions des parties molles, à la notion de transfusion sanguine et au décès maternel, la comparaison entre les primipares et les multipares n'avait pas montré de différence statistiquement significative (p > 0,05).

**Tableau 1:** Caractéristiques sociodémographiques des accouchées

Variables	Cas (n=586)		Témoins (n=1286)		OR [IC95%]	p
	n	%	n	%		
**Âge**						
<20 ans	173	(30,46)	46	(3,58)	11,27 [7,98-15,91]	<0,00001
20-34 ans	386	(67,96)	1157	(89,97)	1,00	
≥35 ans	9	(1,58)	83	(6,45)	0,32 [0,16-0,65]	0,0014
**Statut matrimonial**						
Seule	52	(9,15)	17	(1,32)	7,62 [4,36-13,30]	<0,00001
En union	516	(90,85)	1269	(98,68)	1,00	
**Nombre des CPN**						
0	117	(20,60)	285	(22,16)	0,86 [0,65-1,14]	0,3434
1-3	273	(48,06)	626	(48,68)	0,91 [0,73-1,15]	0,5030
≥4	178	(31,34)	375	(29,16)	1,00	
**Niveau de scolarité**						
Non scolarisée	27	(4,75)	86	(6,69)	0,70 [0,45-1,10]	0,1591
Primaire	49	(8,63)	99	(7,70)	1,11 [0,77-1,60]	0,6154
Secondaire	395	(69,54)	891	(69,28)	1,00	
Supérieur	97	(17,08)	210	(16,33)	1,04 [0,79-1,36]	0,8170
Profession						
Sans emploi	477	(83,98)	1125	(87,46)	1,00	
Travailleuse	41	(7,22)	140	(10,90)	0,69 [0,45-0,95]	0,0454
Élève/Étudiante	50	(8,80)	21	(1,64)	5,61 [3,33-9,45]	<0,00001

**Tableau 2:** Morbidité et mortalité maternelles

Variables	Cas		Témoins		OR [IC95%]	p
	n	%	n	%		
**Mode d’admission**						
Transférée	36	(6,34)	61	(4,74)	1,35 [0,88-2,07]	0,1907
Non transférée	532	(93,66)	1225	(95,26)	1,00	
PA élevée						
Oui	57	(10,04)	71	(5,52)	1,91 [1,32-2,74]	0,0005
Non	511	(89,96)	1215	(94,48)	1,00	
**Hauteur utérine**						
≤34 cm	462	(83,39)	1251	(76,86)	1,00	
>34 cm	92	(16,61)	35	(23,14)	0,66 [0,51-0,86]	0,0021
**État des membranes fœtales**						
Rompues	116	20,42	271	21,07	0,96 [0,75-1,22]	0,7981
Intactes	452	79,58	1015	78,93	1,00	
**Mal présentation fœtale**						
Oui	29	(5,11)	35	(2,72)	1,95 [1,16-3,17]	0,0141
Non	539	(94,89)	1251	(97,28)	1,00	
**Utilisation d’ocytociques**						
Oui	214	(37,68)	294	(22,86)	2,03 [1,64-2,52]	<0,00001
Non	354	(62,32)	992	(77,14)	1,00	
**Mode d’accouchement**						
Césarienne	74	(13,03)	88	(6,84)	2,04 [1,47-2,83]	<0,00001
Voie basse	494	(86,97)	1198	(93,16)	1,00	
Épisiotomie						
Oui	225	39,61	53	4,12	11,89 [8,61-16,43]	<0,00001
Non	343	60,39	1233	95,88	1,00	
**Lésions des parties molles**						
Présentes	44	(7,75)	102	(7,93)	1,03 [0,71-1,48]	0,9657
Absentes	524	(92,25)	1184	(92,07)	1,00	
Éclampsie						
Présente	11	(1,94)	6	(0,47)	4,21 [1,55-11,44]	0,0051
Absente	557	(98,06)	1280	(99,53)	1,00	
**Transfusion**						
Oui	4	(0,70)	7	(0,54)	1,29 [0,37-4,44]	0,9320
Non	564	(99,30)	1279	(99,46)	1,00	
**Issue maternelle**						
Décès	1	(0,18)	3	(0,23)	0,75 [0,07-7,26]	1,0000
Survie	567	(99,82)	1283	(99,77)	1,00	

Les paramètres en rapport avec les nouveau-nés sont présentés dans le Tableau 3. Les proportions des nouveau-nés de faible poids de naissance et des macrosomes étaient respectivement de 10,04 et 3,17% chez les cas contre respectivement 7,31 et 5,29% chez les témoins ; la comparaison de différentes classes de poids de nouveau-nés ne montre pas de différence statistique significative (p>0,05). Sept virgule trente-neuf pourcent de nouveau-nés de cas avaient un score d'Apgar déprimé à la fin de la 5^ème^ minute contre 4,9% de nouveau-nés de témoins et nous avons noté une relation significative entre la dépression néonatale et la primiparité (p = 0,0419); ainsi le risque de dépression néonatale à la 5^ème^ minute est multiplié par 1,55 chez les nouveau-nés issus des primipares (OR=1,55 (1,03-2,32)). La mortalité périnatale était de 4,93% chez les nouveau-nés issus de cas contre 2,80% chez ceux de témoins. L'analyse montre qu'il existe une influence statistiquement significative de la primiparité sur la létalité périnatale (p = 0,0293) signifiant un risque de décès périnatal multiplié par 1,8 fois chez les nouveau-nés des primipares (OR=1,80 (1,08-2,98)).

**Tableau 3:** Paramètres des nouveau-nés

Variable	Cas (n=568)		Témoins (n=1286)		OR [IC95%]	p
	n	(%)	n	(%)		
**Poids de naissance**						
<2500 grammes	57	(10,04)	94	(7,31)	1,38 [0,98-1,95]	0,0799
2500-3999 grammes	493	(86,80)	1124	(87,40)	1,00	
≥4000 grammes	18	(3,17)	68	(5,29)	0,60 [0,35-1,02]	0,0777
**Âge gestationnel**						
<37 SA	61	(15,93)	117	(13,78)	1,21 [0,86-1,70]	0,3005
37-42 SA	282	(73,63)	657	(77,39)	1,00	
>42 SA	40	(10,44)	75	(8,83)	1,24 [0,83-1,86]	0,3489
**Score d’Apgar**						
<7	42	(7,39)	63	(4,90)	1,55 [1,03-2,32]	0,0419
≥7	526	(92,61)	1223	(95,10)	1,00	
**Issue périnatale**						
Décès	28	(4,93)	36	(2,80)	1,80 [1,08-2,98]	0,0293
Survie	540	(95,07)	1250	(97,20)	1,00	

**Figure 2:**
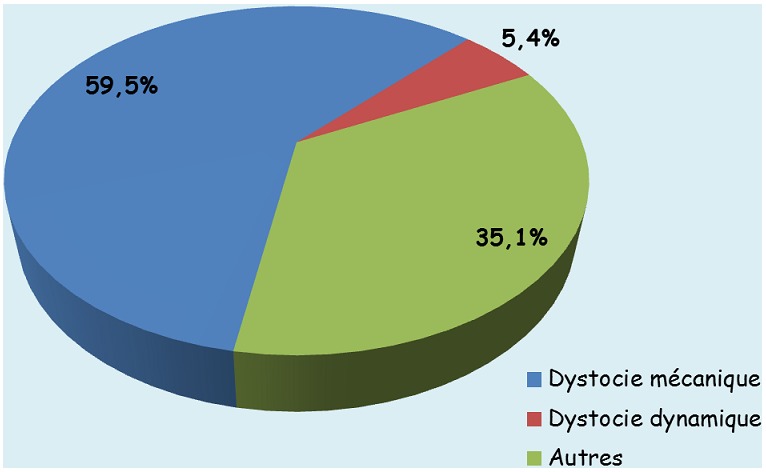
Type d’indications de césariennes chez les primipares (n = 74)

**Figure 3:**
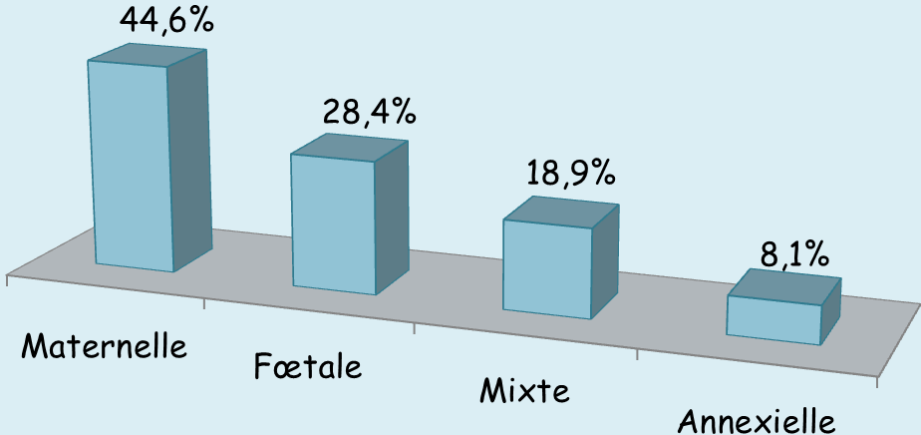
Origines d’indications de césariennes chez les primipares (n = 74)

Des 568 primipares, 74 (13,0%) ont accouché par césarienne. La répartition des indications de césarienne selon le type montre que 64,9% étaient les dystocies (Figure 2). La Figure 3 montre que l’origine maternelle (33/74 soit 44,6%) a dominé les origines d’indications de césarienne suivie de l’origine fœtale (21/74 soit 28,4%). Ni l'âge, ni le statut matrimonial, ni l'état des membranes fœtales, ni l'âge gestationnel n'ont montré une quelconque influence sur la voie d'accouchement chez les primipares. Par contre, nous constatons que l'évacuation obstétricale comme mode d'admission (OR = 9,69 (4,75-19,74)), le manque de suivi de la grossesse (OR=2,57 (1,32-5,01)), la taille 150 cm ou moins (OR = 2,42 (1,04-5,65)), la hauteur utérine supérieure à 34 cm (OR = 2,33 (1,32-4,10)) et la mal présentation fœtale (OR = 6,37 (2,92-13,87)) présentent une association significative avec l'accouchement par césarienne chez les primipares (Tableau 4).

**Tableau 4:** Etude des facteurs influençant l’accouchement par césarienne chez les primipares

Variable	Césarienne		Voie basse		Total	OR [IC95%]	p
Âge	(n=74)		(n=494)		(N=568)		
<20 ans	19	(10,98%)	154	(89,02%)	173	0,79 [0,45-1,38]	0,4967
20-34 ans	52	(13,47%)	334	(86,53%)	386	1,00	
≥35 ans	3	(33,33%)	6	(66,67%)	9	3,19 [0,50-15,53]	0,1163
**Statut matrimonial**	(n=74)		(n=494)		(N=568)		
Seule	9	(17,31%)	43	(82,69%)	52	1,45 [0,67-3,11]	0,4558
En union	65	(12,60%)	451	(87,40%)	516	1,00	
**Mode d’admission**	(n=74)		N=(494)		(N=568)		
Transférée	19	(52,78%)	17	(47,22%)	36	9,69 [4,75-19,74]	<0,00001
Non transférée	55	(10,34%)	477	(89,66%)	532	1,00	
**Nombre de CPN**	(N=74)		(N=494)		(N=568)		
0	25	(21,37%)	92	(78,63%)	117	2,57 [1,32-5,01]	0,0075
1-3	32	(11,72%)	241	(88,28%)	273	1,26 [0,67-2,34]	0,5690
≥4	17	(9,55%)	161	(90,45%)	178	1,00	
**Taille**	(n=59)		(n=411)		(N=470)		
≤150 cm	8	(24,24%)	25	(75,76%)	33	2,42 [1,04-5,65]	0,0355
>150 cm	51	(11,67%)	386	(88,33%)	437	1,00	
**Hauteur utérine**	(n=73)		(n=481)		(N=554)		
≤34 cm	52	(11,26%)	410	(88,74%)	462	1,00	
>34 cm	21	(22,83%)	71	(77,17%)	92	2,33 [1,32-4,10]	0,0046
**Etat des membranes**	(n=74)		(n=494)		(N=568)		
Rompues	20	(17,24%)	96	(82,76%)	116	1,53 [0,88-2,68]	0,1749
Intactes	54	(11,95%)	398	(88,05%)	452	1,00	
**Malprésentation fœtale**	(n=74)		(n=494)		(N=568)		
Oui	13	(44,83%)	16	(55,17%)	29	6,37 [2,92-13,87]	<0,00001
Non	61	(11,32%)	478	(88,68%)	539	1,00	
**Âge gestationnel**	(n=42)		(n=341)		(N=383)		
<37 SA	5	(8,20%)	56	(91,80%)	61	0,65 [0,24-1,74]	0,5230
37-42 SA	34	(12,06%)	248	(87,94%)	282	1,00	
>42 SA	3	(7,50%)	37	(92,50%)	40	0,59 [0,17-2,02]	0,5613

## Discussion

Notre étude rapporte une fréquence de primiparité de 19,9%. La fréquence des femmes ayant leur première expérience d'accouchement va en augmentant au fil du temps dans le monde entier et varie d'un pays à l'autre vu que le contexte de chaque pays et de chaque société diffère. Notre fréquence est supérieure à celle rapportée par Okunade qui trouvait 15,3% [7]. Par contre, elle est inférieure à celles enregistrées par d'autres auteurs: 27,3% pour Danish [2], 37,7% pour Ojiyi [6] et 40% pour Latif [11]. Cette fréquence élevée dans ces études pourrait s'expliquer par une faible utilisation de services de santé maternelle par les multipares. Dans certains pays en développement, les femmes ayant une grande parité ont tendance à compter sur leur expérience de grossesses antérieures estimant qu´elles savent déjà à quoi s'attendre pendant la grossesse et l'accouchement; et plusieurs études ont montré que la multiparité était associée à l'accouchement à domicile [12–14]. Comme le premier accouchement est habituellement connu pour être plus difficile et que la primipare est à sa première expérience d'accouchement, il est probable qu´elle soit plus susceptible de demander de l´aide et des conseils professionnels [14]. Notre étude multicentrique a permis de décrire le profil sociodémographique des primipares à Lubumbashi. Les primipares dans notre milieu sont jeunes, vivant seules (célibataires) et élèves/étudiantes. Elles avaient 11,27 fois plus de chance d'être adolescentes par aux multipares. Nous avons également trouvé que l'âge moyen des primipares (22,3 ± 4,5 ans) était statistiquement inférieur à celui des multipares (26,8±4,7ans). Ce constat a été fait par d'autres auteurs [7]. Il n'est pas surprenant que les femmes portant leur première grossesse sont généralement du groupe d'âge plus jeune car la parité évolue proportionnellement avec l'âge. Comme constaté dans les études antérieures [7], notre étude montre que les primipares n'ont pas présenté un niveau de scolarité différent de celui des multipares. Par contre, Dedecker, dans son étude menée à l'Île de la Réunion, trouvait que les adolescentes primipares avaient un bas niveau de scolarité et ceci pourrait s'expliquer par leur jeune âge mais peut-être aussi par une sortie prématurée du système scolaire une fois leur enfant né [15]. Une étude brésilienne a montré que moins de 30% des mères adolescentes primipares poursuivent leurs études contre 68% de celles qui choisissent l'interruption volontaire de grossesse [16].

Contrairement aux autres études [17,18], notre étude ne montre pas de différence significative entre les primipares et les multipares quant aux taux de fréquentation de services de soins prénataux ainsi qu'au nombre moyen de consultations prénatales. Selon l'OMS, dans les pays en développement, les jeunes femmes enceintes se présentent souvent tardivement aux CPN (dans le deuxième ou troisième trimestre de grossesse) ou ne se présentent même pas pour des soins prénatals. Les raisons évoquées pour cette apathie vers les services de soins prénatals comprennent: (a) l'ignorance de l'importance des soins prénatals (surtout chez les non inscrites), (b) le manque de soutien familial ou social (surtout chez les célibataires), (c) la non disponibilité des services de soins prénatals, (d) la pauvreté, (e) des remarques désagréables des agents de santé vers les non mariées qui sont enceintes. En outre, elles tentent de se soustraire du regard du public puisque certaines cliniques manquent d'intimité [19], mais aussi la crainte de dépistage du VIH pour certaines de ces jeunes femmes [20]. Par contre, au Soudan, une étude rapportait une association significative entre la multiparité et la faible fréquentation des services de soins prénataux et expliquant que les multipares ont tendance à répondre à leur expérience des grossesses antérieures et ne ressentent pas le besoin de contrôles prénatals, estimant qu'elles savent déjà à quoi s'attendre pendant la grossesse ou l'accouchement [14]. Dans la présente étude, les complications obstétricales et les issues de la grossesse chez les mères primipares ont été comparées à celles des mères multipares pendant la période intra- et postpartales. Les résultats montrent, chez les primipares par rapport aux multipares, un risque élevé de pression artérielle élevée à l'admission, de mal présentation fœtale, d'accouchement par césarienne, de recours à l'utilisation d'ocytociques, d'éclampsie, de dépression néonatale et de mortalité périnatale. Comme dans d'autres études [20–26], cette étude avait montré une association très significative entre la primiparité et les troubles hypertensifs. Comparées aux multipares, les primipares présentaient 1,9 et 4 fois plus de risque respectivement d'avoir une pression artérielle élevée et de faire une éclampsie. Surnommée « maladie de primiparité », la pré-éclampsie était fréquente chez les femmes primipares qui présentent un risque de 2,4 fois plus élevé que chez les multipares tel que le montre une revue de littérature faite par Luo [24]. Ceci suggérerait l'implication d'un facteur immunologique. Il est possible que la tolérance des antigènes fœto-placentaires par l'organisme maternel soit facilitée par l´exposition préalable aux antigènes paternels [27].

La mal présentation fœtale était près de deux fois plus élevée chez les primipares comparativement aux multipares. La primiparité est associée à une présentation anormale du fœtus. Il est décrit que vers le 7^ème^ mois de la grossesse, il y a mutation spontanée du fœtus en siège selon la loi d'adaptation de Pajot (adaptation du contenu au contenant) et à la suite de la modification de forme de l'utérus liée à la formation du segment inférieur [28,29]. Au cours de cette mutation, les membres inférieurs entrent en jeu les premiers puis suivent les fesses ; et suite à la pesanteur et à la paroi utérine non encore bien développée chez la primipare, il en résulte un échec de la culbute physiologique expliquant ainsi la prédominance de la mal présentation fœtale chez la primipare retrouvée aussi bien dans la littérature que dans notre étude [29]. Cependant, contrairement à nos résultats, d'autres auteurs ont trouvé qu'une parité élevée était associée avec une présentation anormale du fœtus [7,30,31]. Le risque d'utilisation d'ocytocique au cours de la parturition était 2,3 fois plus élevé chez les primipares que chez les mulitpares. Nos résultats corroborent avec ceux d'autres auteurs [7–9,32] qui ont trouvé également une association significative entre la primiparité et le recours à l'utilisation d'ocytociques. Plusieurs études ont montré que, comparativement aux femmes multipares, les primipares ont un travail d'accouchement plus long [33–35], sont exposés à un risque accru de complications intra-partales et subissent pratiquement plus d'interventions obstétricales [22,32,36]. Des études ont démontré que l'utilisation d'ocytocique chez les primipares diminuait la durée d'accouchement de 2 heures sans causer des effets néfastes pour le fœtus [37,38]. En ce qui concerne l'épisiotomie, l'on a enregistré une proportion de 39,61% chez les primipares et sa pratique était près de 12 fois plus élevée chez les primipares que chez les multipares. Une étude menée en Amérique Latine avait trouvé que l'épisiotomie était pratiquée 92% des primipares [39]. A Karachi (Pakistan), Hashim rapportait 84% d'épisiotomie chez les primipares [22]. L'épisiotomie est devenue une pratique libérale, bien avant la réalisation d'essais cliniques. Elle a pour objectif la prévention du traumatisme périnéal et ano-rectal. C'est un acte médical largement pratiquée chez les primipares comparativement aux multipares. Selon les chiffres du réseau sentinelle français AUDIPOC, une épisiotomie a été réalisée chez 68% des primipares et 31% des multipares en 2002-2003 [29]. Nous avons enregistré 13,03% de césarienne chez les primipares, ce qui est inférieur aux taux rapportés par d'autres auteurs, variant de 25 à 43,2% [6,40,41]. La littérature souligne que les primipares sont des patients à risque élevé et doivent bénéficier des soins prénatals complets. Ils ajoutent en disant que ces taux de césariennes sont inacceptables vu les implications de la césarienne sur l'avenir de la reproduction de ces patientes, en particulier dans nos régions où la grande taille de la famille est souhaitée.

La présente étude montre que le taux de césarienne était 2,04 fois plus élevé chez les primipares comparativement aux multipares, résultats similaires rapportés par Elrishi (2,6 fois) [40] et par Kiliç (3,86 fois) [42]. Le taux de césariennes élevé chez primipares trouvé dans cette étude serait une conséquence de diverses complications de la grossesse et du travail (éclampsie, mal présentation) retrouvées plus fréquemment chez les primipares que chez les multipares. De plus, les grossesses de primipares âgées sont souvent considérées comme des grossesses précieuses; d'où, certains obstétriciens évitent de prendre plus de risque avec accouchement par voie basse. Les dystocies étaient la principale indication de césarienne (64,9%) chez les primipares de notre série. Ce constat est similaire à celui fait par Elrishi [40]. Les primipares sont plus à risque d'anomalies du travail qui nécessitent des interventions intrapartales à cause des tissus de l'appareil reproducteur qui ont plus de résistance [8]. Selon Dudley [43], il faut plus de force utérine nécessaire pour surmonter la résistance dans le tractus reproducteur ce qui fait que l'utérus tend à être moins efficace pour maintenir les contractions utérines. Alors que chez les femmes multipares, une moindre force utérine est nécessaire parce que les tissus du tractus reproducteur ont été étirés par l'accouchement précédent, ont moins de résistance, et par conséquent le myomètre de la multipare maintient généralement une activité contractile efficace [43]. Ceci peut expliquer pourquoi les dystocies soient une raison commune d'intervention chez les primipares [8]. Nos résultats montrent que la référence à partir d'une autre structure sanitaire, le manque de suivi prénatal, la taille ≤ 150 cm, la hauteur utérine > 34 cm et la mal présentation fœtale sont des facteurs spécifiques qui influençaient significativement l'accouchement par césarienne chez les primipares de notre cadre d'étude. En effet, beaucoup de femmes primipares en particulier les adolescentes non mariées ne suivent pas les CPN et accouchent dans des structures de soins, parfois clandestines, peu équipées et ayant peu de compétences en soins obstétricaux et néonataux d'urgence. Les malades sont souvent alors référées dans les hôpitaux de niveau secondaire ou tertiaire en cas de complication ou lorsque le travail se prolonge, les membranes ayant préalablement été déjà rompues.

Le fait que la hauteur utérine influe sur la césarienne est tout à fait compréhensible car la hauteur utérine > 34 cm, en dehors d'hydramnios, présume que le fœtus a un poids ≥ 4000 grammes et que la macrosomie est directement sanctionnée par la césarienne chez les primipares. En plus, la surdistension utérine diminue l'efficacité de contractions utérines. Bien que le taux de césarienne fût plus élevé chez les primipares mesurant 150 cm ou moins que chez celles de plus de 150 cm, l'étude d'Adeyemi ne montre pas d'association entre la taille et la césarienne chez les primipares. Par contre, il retrouve l'indice de masse corporelle et le poids de naissance comme facteurs de risque de césarienne [5]. S'agissant paramètres néonataux, la naissance d'un nouveau-né de faible poids a été retrouvé plus fréquente chez les primipares dans des études antérieures [20,44]. Les résultats de notre étude et de ceux rapportés par Ilunga [45] renforcent bien ces études antérieures. Quant à la dépression néonatale (score d'Apgar < 7) était significativement associée à la primiparité dans notre étude et dans les études antérieures [7,20,22]. Le besoin de soins intensifs néonatals qui a été considérablement noté dans le groupe de primipares pourrait être attribué aux taux élevés de complications intrapartales tels que le travail prolongé, les dystocies et la césarienne qui se sont produits plus parmi les primipares. Enfin, nous avons trouvé que le décès périnatal était 1,8 fois plus élevé chez les primipares que chez les multipares ; ceci serait secondairement dû à la détresse fœtale aiguë et au mauvais score Apgar.

## Conclusion

Cette étude montre que l'accouchement de la primipare reste un problème obstétrical à Lubumbashi. D'où l'amélioration de la santé du couple mère-enfant lors de l'accouchement chez la primipare passe par l'élaboration des protocoles de prise en charge des accouchements adéquats.

### Etat des connaissances actuelle sur le sujet

- L'accouchement chez la primipare constitue un problème majeur de santé publique en République Démocratique du Congo;
- L'accouchement chez la primipare portent un très haut risque de morbidité et mortalité lié aux caractéristiques physiologiques et sociologiques des adolescentes.

### Contribution de notre étude à la connaissance

- Aucune étude sur ce sujet n'a déjà été publiée antérieurement sur les facteurs de risque et le pronostic maternel et périnatal de l'accouchement chez la primipare dans notre contexte, à Lubumbashi, République Démocratique du Congo;
- L'étude proposée est la première étude globale et multicentrique dans notre ville voire dans notre pays, intégrant une analyse factorielle permettant d'évaluer le pronostic maternel et périnatal dans notre contexte.

## Conflits d’intérêts

Les auteurs ne déclarent aucun conflit d'intérêt.
